# Assessing and Broadening Genetic Diversity of *Elymus sibiricus* Germplasm for the Improvement of Seed Shattering

**DOI:** 10.3390/molecules21070869

**Published:** 2016-07-01

**Authors:** Zongyu Zhang, Junchao Zhang, Xuhong Zhao, Wengang Xie, Yanrong Wang

**Affiliations:** The State Key Laboratory of Grassland Agro-ecosystems, College of Pastoral Agriculture Science and Technology, Lanzhou University, Lanzhou 730020, China; zhangzymail@163.com (Z.Z.); 13669621907@163.com (J.Z.); zhaoxh14@lzu.edu.cn (X.Z.)

**Keywords:** *Elymus sibiricus*, EST-SSRs, genetic diversity, seed shattering

## Abstract

Siberian wild rye (*Elymus sibiricus* L.) is an important native grass in the Qinghai-Tibet Plateau of China. It is difficult to grow for commercial seed production, since seed shattering causes yield losses during harvest. Assessing the genetic diversity and relationships among germplasm from its primary distribution area contributes to evaluating the potential for its utilization as a gene pool to improve the desired agronomic traits. In the study, 40 EST-SSR primers were used to assess the genetic diversity and population structure of 36 *E. sibiricus* accessions with variation of seed shattering. A total of 380 bands were generated, with an average of 9.5 bands per primer. The polymorphic information content (PIC) ranged from 0.23 to 0.50. The percentage of polymorphic bands (P) for the species was 87.11%, suggesting a high degree of genetic diversity. Based on population structure analysis, four groups were formed, similar to results of principal coordinate analysis (PCoA). The molecular variance analysis (AMOVA) revealed the majority of genetic variation occurred within geographical regions (83.40%). Two genotypes from Y1005 and ZhN06 were used to generate seven F_1_ hybrids. The molecular and morphological diversity analysis of F_1_ population revealed rich genetic variation and high level of seed shattering variation in F_1_ population, resulting in significant improvement of the genetic base and desired agronomic traits.

## 1. Introduction

*Elymus sibiricus* L., commonly known as siberian wildrye, is a perennial, cold-season, self-pollinating, and allotetraploid grass with the StStHH genome constitution (2*n* = 28) [1]. Indigenous to northern Asia, *E. sibiricus* germplasm are especially rich and diverse in north China, where it is distributed primarily in Qinghai-Tibet Plateau, Inner Mongolia, Sichuan, Xinjiang, and Gansu Provinces [2]. *E. sibiricus* has been widely grown for pasture and hay, owing to its excellent stress tolerance, good forage quality and adaptability to local environments, and it therefore plays an important role in animal husbandry and sustenance in North China [3].

As an economically important species, *E. sibiricus* is difficult to grow for commercial seed production since seed shattering can cause up to 80% yield losses if harvesting is delayed [4]. The provinces of Qinghai and Sichuan, China, where the majority of *E. sibiricus* seed (2,400,000 kg) is produced each year, accounts for over 90% of total seed yield. However, the average seed production of *E. sibiricus* is only 690 kg∙ha^−1^ (China Grass Internet). To reduce seed shattering and enhance seed production, one of the most important approaches is to explore genetic diversity.

Morphologically and genetically diverse germplasm is a potentially valuable source for the improvement of desired agronomic traits such as seed yield, quality and stress tolerance [5]. To broaden the genetic base of *E. sibiricus* germplasm, one important strategy is to develop novel breeding lines by using genetically and phenotypically diverse germplasm to cross with the adapted cultivars. Generally, these resynthesized breeding lines are genetically diverse from inbred line/cultivar [6], and hybrids from two parents with distant genetic base might have higher heterosis [7]. Recent research has revealed wide variation in seed shattering among wild *E. sibiricus* germplasm from Qinghai-Tibet Plateau [2,8] and suggested these wild germplasm have great potential for the improvement of seed shattering. Additionally, a previous report showed that genetic distance between germplasm can be a predictor of combining ability [9]. It is, therefore, important to study the genetic diversity of *E. sibiriucs* germplasm with variation of seed shattering from its primary distribution area for improving our understanding of breeding materials and developing more efficient conservation and breeding strategies.

The development of neutral molecular markers has made it fast, reliable and accurate to reveal the genetic diversity of germplasm. Compared with other molecular markers like inter simple sequence repeat (ISSR), sequence-related amplified polymorphism (SRAP), and start codon targeted (SCoT) etc, EST-SSRs are highly polymorphic, abundant and are accessible to research in laboratories via published primers sequences. What is more, EST-SSRs have a higher level of transferability across related species than genomic-SSRs because EST-SSRs originate from the transcribed regions in genomes and possess conserved sequences among homologous genes [5]. Along with the development of next-generation sequencing, transcriptome sequencing has also become an efficient method to identify large EST sequences and develop EST-SSR markers [10]. To date, EST-SSRs have been widely used for genetic diversity [11], genetic mapping [12], and DNA fingerprinting [13].

The pattern of genetic variability of the available germplasm substantially affects the choice of breeding materials and the success of plant breeding programs. The objectives of the present study were to (i) compare the genetic diversity and relationship among *E. sibiriucs* accessions from North China; (ii) broaden the genetic diversity of *E. sibiricus* by crossing two genetically and morphologically diverse genotypes and assess genetic variation of the hybrid population.

## 2. Results

### 2.1. Seed Shattering Degree of 36 E. sibiricus Accessions

**Figure 1:**
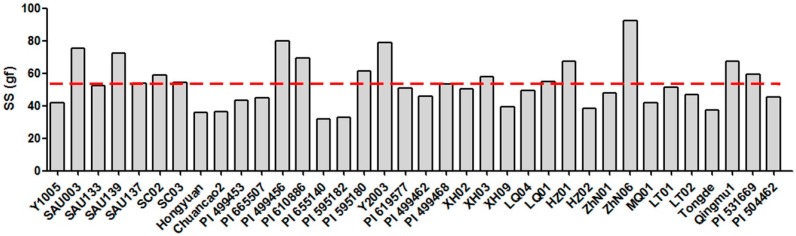
Seed shattering degree (SS) of 36 *E. sibiricus* accessions. The red dotted line represents the average value of seed shattering.

The BTS value among 36 *E. sibiricus* accessions varied from 31.86 gf (PI655140) to 92.34 gf (ZhN06), with an average of 53.28 gf. Sixteen accessions had a relatively low seed shattering degree with BTS more than the average value of seven accessions including four wild accessions (PI655140, PI595182, HZ02 and XH09) and three cultivars (Hongyuan, Chuancao2 and Tongde) had a relatively high seed shattering degree with the BTS value of less than 40 gf. The other 13 accessions had a moderate seed shattering degree (Figure 1).

### 2.2. Polymorphism of EST-SSR Markers and Genetic Relationships of 36 E. sibiricus Accessions

Furthermore, we analyzed the genetic diversity and variation of 36 *E. sibiricus* accessions with variation of seed shattering degree (Table 1). One hundred EST-SSR primers selected from *Elymus*, *Pseudoroegneria* and *Leymus* EST database, and 112 novel *E. sibiricus* EST-SSR markers developed by transcriptome sequencing were chosen to conduct the primers′ screening. Finally, 40 EST-SSR primers that successfully amplified clear and stable bands were selected to evaluate the genetic diversity of these 36 accessions (Table 2). The 40 primers generated 380 bands, 331 of which were polymorphic. The percentage of polymorphism (P) was 87.11%. The total bands (T) per primer ranged from 2 (ES-405) to 22 (Elw5616s393) with 9.5 bands per primer. Across the 36 accessions, the polymorphic information content (PIC) values ranged from 0.23 (ES-22 and ES-125) to 0.50 (Elw2698s152 and Elw2807s159, etc.) with an average of 0.44, suggesting a high level of polymorphism.

**Figure 2:**
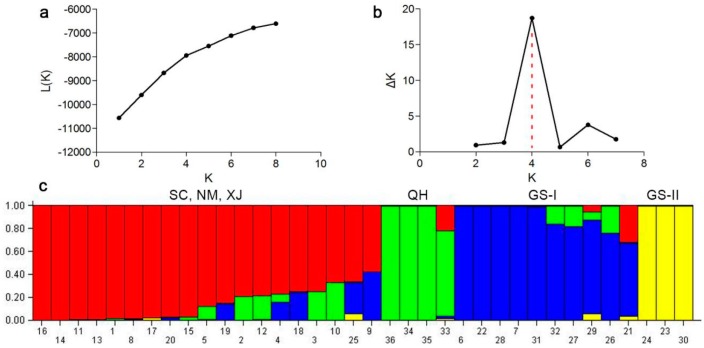
Four groups of 36 *E. sibiricus* accessions inferred from STRUCTURE analysis and the description of detected the optimum value of K by using graphical method. (**a**) Mean L (K) over 20 runs for each K value; (**b**) Maximum delta K (△K) values were used to determine the uppermost level of structure for K ranging from 2 to 7, here K is four and four clusters; (**c**) The vertical coordinate of each group indicates the membership coefficients for each accessions. Red zone: SC, NM and XJ; Green zone: QH; Blue zone: GS-I; Yellow zone: GS-II.

The population structure of the 36 accessions was investigated using the Hardy-Weinberg Equilibrium by using STRUCTURE V2.3.4 software. Based on maximum likelihood and delta K (ΔK) values, the number of optimum groups was four (Figure 2). Among them, 18 accessions from Sichuan, Inner Mongolia and Xinjiang and one from Gansu were assigned to group 1 (SC, NM, XJ); four accessions from Qinghai were assigned to group 2 (QH); eight accessions from Gansu and two from Sichuan were assigned to group 3 (GS-I); three accessions from Gansu were assigned to group 4 (GS-II). Among 36 accessions, Y1005 and ZhN06 showed the largest genetic distance (0.6752). The results of genetic structure showed that there was not a strong relationship between the genetic structure and the geographical origin. For example, SC02 and SC03 from Ruoergai, Sichuan were assigned to group 3, showing close genetic relationship with accessions from Gansu.

**Figure 3:**
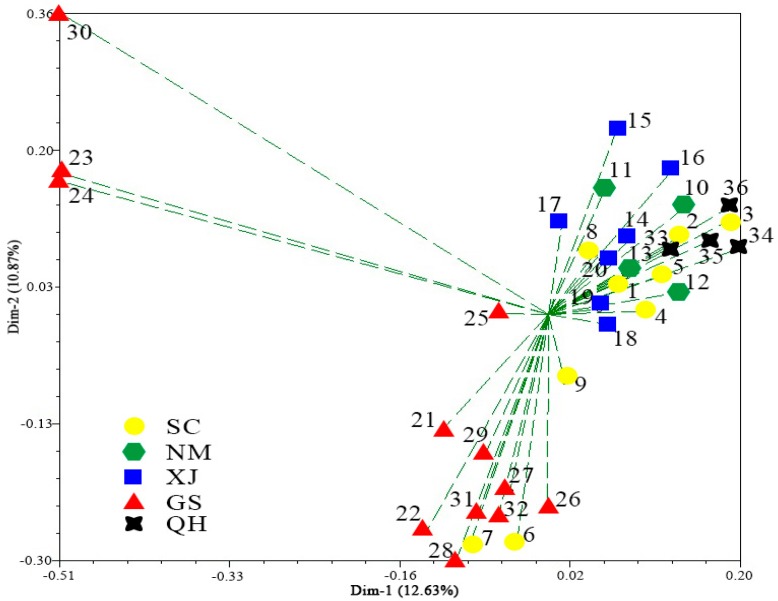
Principal coordinates analysis for the first and second coordinates estimated for EST-SSR markers using Jaccard′s genetic similarity matrix for 36 *E. sibiricus* accessions.

The principal coordinate analysis (PCoA) showed about 31.52% of the total variation was described by the first three PCo (Figure 3). The majority of accessions from GS and two from SC (SC02 and SC03) shared the same group, three accessions from GS were assigned to one group, the remaining of SC as well as NM, XJ and QH were assigned to a mixed group. The results of PCoA analysis were similar to structure analysis, indicating the reliability of the results.

Results of POPGENE analysis showed high genetic diversity between geographic regions (Table 3). The values of NPB ranged from 70 (QH) to 299 (GS), with an average of 177.4. The PPB values ranged from 32.71% to 86.17%, with an average of 63.13%. The Shannon information index of diversity (I) values ranged from 0.0946 to 0.3594, with an average of 0.2301. The Nei′s genetic diversity (H) values ranged from 0.0623 to 0.2315, with an average of 0.1510. The observed number of alleles values (Na) ranged from 1.1842 to 1.7868, with an average of 1.4668. Generally, among five geographic regions, GS exhibited the highest level of variability (PPB = 86.17%, I = 0.3594, H = 0.2315, Na = 1.7868), whereas the group QH exhibited the lowest of variability (PPB = 32.17%, I = 0.0946, H = 0.0623, Na = 1.1842). AMOVA analysis showed a significant (*p* < 0.001) genetic difference among the five regions. A larger proportion variation (83.40%) was apportioned within geographic regions and 16.60% was apportioned between geographic regions (Table 4). The genetic identity among five geographic regions ranged from 0.6170 (between NM and QH) to 0.9552 (between NM and XJ) with an average of 0.8218 (Table 5).

### 2.3. Genetic and Phenotypic Variation of Hybrid Population

Two parental genotypes: Y1005-1 (moderate seed shattering degree) and ZhN06-1 (lowest seed shattering degree)were selected as parents to produce seven F_1_ individuals by hand pollination, because they had the highest genetic distance and contrasting seed shattering degree. The phenotypic variation and genetic diversity of the hybrid population and their parents were studied using 12 phenotypic traits and EST-SSR markers. Table 6 showed mid-parent heterosis (MPH), higher-parent heterosis (HPH), and coefficient of variation (CV) of the 12 traits for hybrid population and their parents. The greatest variation was found for 1000-seed weight (CV = 33.11%) and seed shattering (SS) (CV = 32.98%), followed by flag leaf length, flag leaf width, tiller number, leaf length, culm diameter, leaf width, plant height, culm number, awn length and panicle length. Some phenotypic traits of the hybrids showed evidence for significant heterosis, including flag leaf length (MPH = 80.9%, HPH = 80.4%), seed shattering (MPH = 51.1%, HPH = 8.1%), leaf length (MPH = 48.4%, HPH = 32.0%) and flag leaf width (MPH = 44.0%, HPH = 23.5%). Whereas the heterosis of tiller number, plant height, 1000-seed weight and culm diameter was lower than that of other phenotypic traits, some of them showed negative heterosis for F_1_ hybrids.

The high degree of genetic variation found in morphological traits is in accord with the genetic variability (Table 7). The 40 EST-SSR primers amplified 257 bands (P = 59.92%), with 6.4 bands per primer. PIC ranged from 0.00 to 0.44, with the average of 0.20. The number of bands exclusively present in F_1_ lines (BEPF) (8.44%) is higher than bands exclusively present in parents (BEPP) (1.95%). 20.78% bands were shared by Y1005-1 and F_1_ lines, whereas 26.62% bands were shared by ZhN06-1 and F_1_ lines. These results showed that ZhN06-1 may have a higher heritability than Y1005-1.

**Figure 4:**
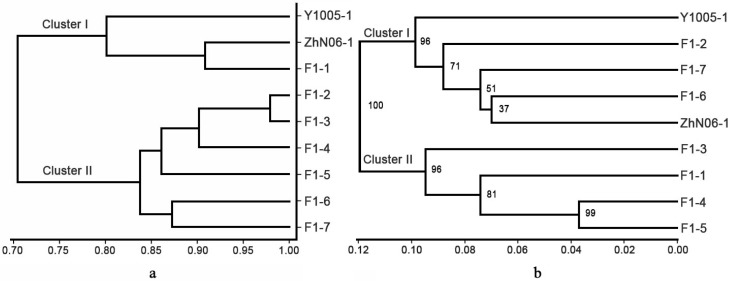
Dendrograms of the parents and F_1_ generations of *E. sibiricus* using UPGMA from phenotypic data (**a**) and marker-based data (**b**). The numbers marked on the branches (**b**) are bootstrap values (%) out of 1000 bootstrapping.

The clustering analysis based on phenotypic traits showed two major groups (Figure 4a). Cluster I contained Y1005-1, ZhN01-1 and F_1_-1. Cluster II included the other six F_1_ lines, among them F_1_-2, F_1_-3 and F_1_-4 were clustered together in a major subgroup with F_1_-5, while F_1_-6 and F_1_-7 were in a separate subgroup. When compared with the phenotypic-based dendrogram, marker-based cluster revealed poor correlation with morphological characteristics (Figure 4b). Cluster I consisted of Y1005, F_1_-2, F_1_-7, F_1_-6 and ZhN06. Other four F_1_ lines were grouped into cluster II.

## 3. Discussion

### 3.1. Genetic Diversity of E. sibiricus

Genetic diversity is the foundation of species diversity and a crucial precursor in the study of any species, because its quantity and distribution have an effect on the evolutionary and breeding potential of species or populations [14]. As an important forage grass in North China, *E. sibiricus* possesses great morphological and genetic variation [15]. However, recent research has showed that global climate warming and excessive grazing threaten the productivity and growth of *E. sibiricus*, causing losses of genetic diversity [16]. It is, therefore, necessary to evaluate the level and distribution of genetic variability for effective exploitation and utilization of *E. sibiricus*. Former studies have assessed *E. sibiricus* accessions and populations of different origins using some molecular markers, including ISSR [17], SRAP [18], SCoT [19] and EST-SSR [2]. Each study found high genetic diversity within accessions or populations. Similar genetic diversity level (87.11%) was found in this study, which might be due to the diverse geographic origins of materials tested. Among five geographic regions, GS has higher genetic diversity (86.17%) than the other four regions: SC, XJ, NM and QH. Accessions from QH revealed the highest genetic distance when compared with other populations. Previous studies showed that environment parameters such as latitude, longitude and altitude are highly correlated with the magnitude and distribution of genetic diversity [3]. The wide geographical range of five *E. sibiricus* populations studied may have contributed to the difference of genetic diversity. Sample size is also an important factor affecting the measurement of genetic diversity [2]. There was a positive correlation between sample size and genetic diversity [19]. In this study, small sample size from some geographic regions (e.g., four accessions from QH) may have resulted in a lower estimate of genetic diversity.

Typically, self-pollinating species possess relatively less within-population genetic variability than out-crossing species [20]. In this study, 83.40% of the genetic variance was apportioned within geographic regions, similar to values previously reported for *E. sibiricus* [2,3,18] and other self-pollinating *Elymus* species. For instance, Stevens et al. [21] found 85.0% within-population variation by analyzing four *E. trchycaulus* populations using SSR markers. Many factors previously reported can affect the pattern of genetic variability such as gene mutation, genetic drift, selection, gene flow, reproduction mode and population size [2,22,23,24]. In this study, genetic divergence may be more related to complex eco-geographical factors within the *E. sibiricus* distribution area.

### 3.2. Broadening Genetic Diversity for Seed Shattering Improvement

Like most native grasses, *E. sibiricus* is difficult to grow for commercial seed production, since seed shattering causes large yield losses during harvest. A major limitation of plant improvement program is the lack of plant materials exhibiting genetic variation for traits of interest [25]. The challenge that exists for plant development is to maintain the genetic diversity within a species while improving desired traits that enable plant materials to perform well. To broaden the genetic diversity of *E. sibiricus* for future breeding improvement programs, two parental genotypes (Y1005-1 and ZhN06-1) with genetic difference sand contrasting seed shattering habits were selected to produce F_1_ lines. Our results showed seed shattering degree in hybrid population ranged from 68.2 gf (F_1_-1) to 143.4 gf (F_1_-6), with an average of 97.2 gf. Three F_1_ individuals (F_1_-4, F_1_-6 and F_1_-7) had lower seed shattering degree than low seed shattering parent ZhN06-1 (97.2). Thus, these individuals could be used as breeding materials for developing low seed shattering cultivars in the future. Except for seed shattering, other traits such as flag leaf length and width also showed the positive heterosis. Our results confirmed that some morphological traits of *E. sibiricus* could be improved by means of hybridization. When compared with the phenotypic-based dendrogram, marker-based cluster revealed poor correlation with morphological characteristics. The phenotypic-based dendrogram using limited morphological data could be affected by environment factors. In comparison, a marker-based cluster is more efficient and allows genetic diversity analysis using any physiological stage or tissue, suggesting its potential in analyzing genetic diversity and relationship of *E. sibiricus*.

Based on our results, the genetic diversity of hybrid population is 59.92%. Furthermore, 8.44% and 1.95% of polymorphic bands were exclusively present in F_1_ lines and parents, respectively. These gained and missed bands were considered as polyploidization-induced rearrangements within coding regions. Hybridization of more genomes with different sizes and compositions in a single nucleus followed by chromosome doubling can induce several types of genomic modifications and rearrangement in the hybrids [26,27]. These new rearranged bands might be associated with effects of heterosis and contribute to surprisingly low seed shattering in the hybrids. However, whether these novel bands were responsible for new genes associated with seed shattering or other important traits is still not clear. In the future, molecular markers combined with sequence data might provide new evidence.

## 4. Materials and Methods

### 4.1. Plant Materials

A total of 36 *E. sibiricus* accessions were used in the study, comprising wild collections, breeding lines, cultivars, and cultivated types (Table 1). Seeds of these accessions were obtained from National Plant Germplasm System (NPGS, USA), Lanzhou University, Sichuan Agricultural University and Sichuan Academy of Grassland Science. All accessions were grouped into five geographic regions: SC (Sichuan), NM (Inner Mongolia), XJ (Xinjiang), GS (Gansu) and QH (Qinghai) based on their origin and physical-geographical regionalization. Moreover, eight F_1_ lines derived from a pair cross between two parental genotypes: Y1005-1 and ZhN06-1 were also used for genetic diversity analysis (Table 2).

### 4.2. DNA Extraction and PCR Amplification

Twenty individuals of each accession were sampled for the extraction of bulked DNA. Leaf tissues were collected from young plants, and were lyophilized for DNA extraction using a modified cetyltrimethyl ammonium bromide (CTAB) method [28]. DNA concentration and quality were determined using a Nanodrop spectrophotometer (NanoDrop Products, Wilmington, DE, USA) and agarose gel electrophoresis. Finally, the DNA samples were diluted to 25 ng/µL and stored at −20 °C prior to PCR amplification.

A total of 212 EST-SSR primers from different resources were used for genotyping, of which 100 EST-SSR markers were previously developed from *Elymus* (Elw hereafter), *Pseudoroegneria* (Ps hereafter) and *Leymus* (Lt hereafter) EST database [29,30,31] and 112 novel *E. sibiricus* EST-SSR markers were developed by transcriptome sequencing [32]. The DNA samples of 5 accessions with different geographical origins were used for primer screening. Then 40 EST-SSR primers that successfully amplified and produced clear and stable bands of the expected size by PCR amplification were used in the final analysis (Table 3). The PCR amplification and SSR genotyping were carried out as described by Xie et al. [2] and Zhou et al. [32]. Amplification fragments were then separated on 6% denatured polyacrylamide gels electrophoresis (PAGE). The resulting gel was stained by AgNO_3_ solution, and photographed by a digital camera (D7000, Nikon, Tokyo, Japan).

### 4.3. Phenotypic Traits Measurement

The seeds of F_1_ lines and their parents were germinated in plastic boxes with moistened blotter paper at room temperature. After germination seedlings were grown in a greenhouse under a 25/15 °C day/night temperature regimes until they were 8 weeks old. Then they were transplanted to field plots in the research farm, Yuzhong, Gansu, China (latitude 35°34′ N, longitude 103°34′ E, elevation 1720 m). Plants were spaced 0.5 m within rows and 1 m between rows. A total of 12 phenotypic traits, including seed shattering (SS), plant height (PH), leaf length (LL), leaf width (LW), flag leaf length (FLL), flag leaf width (FLW), culm diameter (CD), culm number (CN), tiller number (TN), panicle length (PL), awn length (AL) and 1000-seed weight (1000-SW) were measured using the methods described by Zhao et al. [8]. Seed shattering degree of *E. sibiricus* accessions was determined by measuring pedicel breaking tensile strength (BTS), which is inversely proportional to shattering degree. Thirty randomly chosen spikelets of each plant were examined at 28 days after heading, and their average BTS values were calculated. The heterosis of hybrids were estimated on mid-parent values and high-parent value using the following formula: mid-parent heterosis (%) = (F_1_ − MP)/MP × 100%, higher-parent heterosis (%) = (F_1_ − HP)/HP × 100 %, where F_1_ is the mean of the hybrids, MP is the mean of parents, HP is the value of higher parent [33].

### 4.4. Data Analysis

The amplified bands were scored as present (1) or absent (0), and only reproducible bands were considered. The resulting present/absent data matrix was analyzed using POPGENE 32 Version 1.31 [34]. Number of polymorphic band (NPB), percentage polymorphic band (PPB), Shannon information index of diversity (I), Nei′s gene diversity (H), and observed number of alleles (Na) and polymorphic information content (PIC) were used to evaluate genetic diversity. PIC was calculated for each primer according to the formula: PIC = 1 − p^2^ − q^2^, where *p* is frequency of present band and q is frequency of absent band [35]. The Analysis of Molecular Variance (AMOVA) was used to partition the total EST-SSR variation into within populations and among populations [36]. The input files for POPGENE and AMOVA were prepared with the aid of DCFA1.1 program written by Zhang and Ge [37]. Population structure of the 36 *E. sibiricus* accessions was analyzed using STRUCTURE v2.3.4 software with the ′admixture mode′, burn-in period of 10,000 iterations and a run of 100,000 replications of Markov Chain Monte Carlo (MCMC) after burn in [38]. For each run, 10 independent runs of STRUCTURE were performed with the number of clusters (K) varying from 1 to 8. Mean L (K) and delta K (ΔK) were estimated using the method described by Evanno et al. [39], maximum likelihood and delta K (ΔK) values were used to determine the optimum number of groups. A principal coordinate analysis (PCoA) was constructed based on Jaccard′s genetic similarity matrix using DCENTER module in NTSYS (version 2.10) [40]. A dendrogram was constructed using the GenStat (version 17.1) and free tree + tree view (version 1.6.6 for Windows) software. The phenotypic data were analyzed using SPSS software (SPSS, version 22 for Windows, SPSS Inc., Chicago, IL, USA).

## 5. Conclusions

This study showed a high level of genetic diversity and a clear population structure of 36 *E. sibiricus* accessions from its primary distribution area in China. The finding that larger variation existed within geographical regions will provide a guideline for the collection and conservation of *E. sibiricus* germplasm. More genetic variation of the species can be captured when sampling a larger number of plants from special eco-geographical regions. Meanwhile, cross breeding is an effective way to obtain more genetic and phenotypic variation. F_1_ lines of *E. sibiricus* exhibited a higher genetic variation in the major agronomic traits. In addition, some F_1_ lines showed obvious heterosis over parents, especially in seed shattering performance. These hybrids could be used as important genetic resources for genetic improvement of *E. sibiricus* in future breeding improvement programs.

## Figures and Tables

**Table 1:** *E. sibiricus* accessions used in the study.

Population	Code	Accession	Origin	Status
SC	1	Y1005	Sichuan, China	Wild
SC	2	SAU003	Kangding, Sichuan, China	Wild
SC	3	SAU133	Aba, Sichuan, China	Wild
SC	4	SAU139	Kangding, Sichuan, China	Wild
SC	5	SAU137	Aba, Sichuan, China	Wild
SC	6	SC02	Ruoergai, Sichuan, China	Wild
SC	7	SC03	Ruoergai, Sichuan, China	Wild
SC	8	Hongyuan	Hongyuan, Sichuan, China	Breeding line
SC	9	Chuancao2	Hongyuan, Sichuan, China	Cultivar
NM	10	PI 499453	Inner Mongolia, China	Wild
NM	11	PI 665507	Inner Mongolia ,China	Wild
NM	12	PI 499456	Inner Mongolia, China	Cultivated
NM	13	PI 610886	Inner Mongolia, China	Wild
XJ	14	PI 655140	Xinjiang, China	Wild
XJ	15	PI 595182	Xinjiang, China	Wild
XJ	16	PI 595180	Xinjiang, China	Wild
XJ	17	Y2003	Xinjiang, China	Wild
XJ	18	PI 619577	Xinjiang, China	Wild
XJ	19	PI 499462	Xinjiang, China	Wild
XJ	20	PI 499468	Xinjiang, China	Cultivated
GS	21	XH02	Xiahe, Gansu, China	Wild
GS	22	XH03	Xiahe, Gansu, China	Wild
GS	23	XH09	Xiahe, Gansu, China	Wild
GS	24	LQ04	Luqu, Gansu, China	Wild
GS	25	LQ01	Luqu, Gansu, China	Wild
GS	26	HZ01	Hezuo, Gansu, China	Wild
GS	27	HZ02	Hezuo, Gansu, China	Wild
GS	28	ZhN01	Zhuoni, Gansu, China	Wild
GS	29	ZhN06	Zhuoni, Gansu, China	Wild
GS	30	MQ01	Maqu, Gansu, China	Wild
GS	31	LT01	Lintan, Gansu, China	Wild
GS	32	LT02	Lintan, Gansu, China	Wild
QH	33	Tongde	Qinghai, China	Cultivar
QH	34	Qingmu1	Qinghai, China	Cultivar
QH	35	PI 531669	Qinghai, China	Wild
QH	36	PI 504462	Qinghai, China	Wild

Note: SC, Sichuan, China; NM, Inner Mongolia, China; XJ, Xinjiang, China; GS, Gansu, China; QH, Qinghai, China.

**Table 2:** Results achieved by *Elymus* (Elw), *Pseudoroegneria* (Ps), *Leymus* (Lt) and *E. sibiricus* (ES) EST-SSR markers, all detected in 36 *E. sibiricus* accessions.

Primer ID	Forward Primer (5′-3′)	Reverse Primer (5′-3′)	T	M	TP	%P	PIC
Elw0300s019	TTCATCCATCCAATTCTAGCACAA	GAAGGAGAAGATGGAATCCTTGAA	8	0	8	100.00	0.49
Elw0669s043	CATCTCACGGCAAGTAAATGAACA	TGCGAGATGGGGTACAATTTTTAT	12	0	12	100.00	0.35
Elw1197s069	ATGGCCGTAACCCTTTACCTGTAT	TTTCAAAGCCTTTCCAAGTGAATC	7	1	6	85.71	0.48
Elw1420s081	GGATAGACCCATGAGCTGACTGAT	CTTTCTCCACAAGTTGAACACAAGA	11	0	11	100.00	0.35
Elw1468s087	TAGCAATAAGTTGCTGCTGCTGTT	CCACCTCTAAATTAATCACCACGAA	11	0	11	100.00	0.47
Elw1675s092	CAGTTAAAATGCTTGTCCAAATGC	CCATGATTGTTCTGTCAAGAAACG	10	1	9	90.00	0.48
Elw2676s146	AATTCGAAAGCTGTGGACTTGTCT	CAATTTTGCTCTCAAGAGAACCGT	10	1	9	90.00	0.48
Elw2698s152	CAAAGCATGTGTAGGCAGTCTTGT	TAACAATGATCAGTTGATCGGACC	9	0	9	100.00	0.50
Elw2807s159	CCCAAGAAGCAAAAGTGAAGTTGA	ATAATTGCTGTAAAACGGCAGGAA	11	0	11	100.00	0.50
Elw2808s160	TTTCATATCCGATACCCAGAAAGC	GGGCGACAAGGGTACTACTAACAA	4	0	4	100.00	0.45
Elw3264s184	TGGACTGCTTTGGGACATAATAGG	CTGAATCATAGCCACCCTGAAAAC	6	0	6	100.00	0.42
Elw3384s187	AGCTCCTGATAGAAAGAGCCATCA	GGCTGCTGGAACTGAAGACAGTA	16	0	16	100.00	0.36
Elw3492s190	TGTTGTTGTTCCAGTTCCAGTCTC	AAAAACAACCACACAAGGTTGTCA	9	2	7	77.78	0.43
Elw3995s226	CTCTAGGGTTTTGGGATTTTAGCC	GTTGTGGAGGTCGGAGAAGGT	7	0	7	100.00	0.49
Elw4419s261	AGGGTGACTTGTCTTTGGGTGTAA	AGTCAGATGAAGGATGGCTGAAAC	8	1	7	87.50	0.50
Elw5447s306	TCCTCAAACTCCTCCTCTCTTCG	GAGGTAAGTCTCGACATCCTCGAC	9	0	9	100.00	0.50
Elw5616s393	TAGTAGCGTGGCACTCCTCTTCTT	GGTACAAACCACCAAAGGTACTGC	22	0	22	100.00	0.42
Elw5627s404	AGATGAAGCTGGTAACCGAGACAG	ATTTCCTCTAATGGAAGCTCTGGC	17	0	17	100.00	0.46
Ps2283	GCCACAACAAGAGAAGACCTTGC	GACCTGCATGATGCTCTCGC	13	0	13	100.00	0.40
Ps261	CTCGAATCCAGCTGAACAATTTCT	AGTCGATCCTCACCTTCATCTCC	9	0	9	100.00	0.45
Ps3447	AGCTTTATGAAGATCGCCACTCAC	CTGCTGCTGCTACCGTTCTTATTT	13	0	13	100.00	0.50
Ps3577	CATCTTGCATATAGCTCCTTCGCT	CTCAAGAAACCCACAATCCAATTC	5	0	5	100.00	0.48
Ps938	TTGCTCCTATGGTTCCACGTAGTT	AAAGTGAAATTCTGCCATCAGAGC	13	1	12	92.31	0.49
Ltc0209	CAGGAACATGAACAGAAGCCTGTC	GTACTGGTCGAACCACCCAAAGT	12	1	11	91.67	0.50
Ltc0096	GCGCACTACCGCCTCTTAGTT	GTCCAGGTAGCACACCTCCG	8	2	6	75.00	0.49
ES-7	CCTCCTCCGTTACCATGTTG	CCCTGCTTTTCCCTCTCTG	4	3	1	25.00	0.27
ES-22	AAGATATCCTGATGCTGGACAAA	GATCAGATCAATAGCTTGAGCG	7	5	2	28.57	0.23
ES-23	CGTACTTGCGCCAGAAGTG	AGGTGTCCATCGAAGGGTC	14	2	12	85.71	0.45
ES-51	GAGCTGAGCTGAGAAGAAAACAG	CACAATCATCTCATCTTCCTTCC	14	0	14	100.00	0.44
ES-97	ACTGTGGGAGAAGGTGAGAGACT	CTTTCCTCCAGCTCATGGTG	8	4	4	50.00	0.49
ES-123	AGCATGAAGCTCGACTGTGAGT	GCGAGTACATCTCGTACTTCTGG	12	4	8	66.67	0.49
ES-125	GAGCATCGACAGATTATTCCTTG	CGAAGGAACCTCTGCAAGAC	5	3	2	40.00	0.23
ES-144	GGTAGTCGTTGACCCAGATGTC	CACATTGTAAACTGGTCCTCCTC	5	0	5	100.00	0.49
ES-231	TAGCTGGTCATGCCTAGGAGTAG	CCAGGTGTCAGGATATAGCAAAA	6	1	5	83.33	0.44
ES-236	TCGCATGCTTATAATCCTTTGAC	TGAGGTCTCTGTCAATACCAACA	6	3	3	50.00	0.35
ES-253	CATCTCTTCAAACTTGGATTGGT	GTGATCTATACCATTGGCCTCAA	12	4	8	66.67	0.46
ES-259	CTCCTCTACCTGTCTGCTGCTA	AGATCGTCGACTACGTCAAGAAG	11	0	11	100.00	0.47
ES-310	CGTAGCAATTCCATTCTATCCAG	TGGTGAGCTAGATTGACACTGAG	9	6	3	33.33	0.33
ES-347	CATGAAGATGATGCGTGTTTTAAT	CCGACTCCTAATTGAACTCGTAA	5	3	2	40.00	0.47
ES-405	AGAGAAAAGGAGATTCTCATCCC	GCTGCTCTGCATCCTACTCTATC	2	1	1	50.00	0.43
Mean			9.5	1.2	8.3	87.11	0.44
Total			380	49	331		

T, total number of amplified bands; M, number of monomorphic bands; TP, total number of polymorphic bands; % P, percentage of polymorphism; PIC, polymorphic information content.

**Table 3:** Genetic variability within five geographic regions of *E. sibiricus*.

POP	NPB	PPB (%)	I	H	Na
SC	204	73.12	0.2650	0.1739	1.5368
NM	137	56.85	0.1947	0.1301	1.3605
XJ	177	66.79	0.2368	0.1570	1.4658
GS	299	86.17	0.3594	0.2315	1.7868
QH	70	32.71	0.0946	0.0623	1.1842
Mean	177.4	63.13	0.2301	0.1510	1.4668

NPB, number of polymorphic band; PPB, percentage of polymorphic bands; I, Shannon information index of diversity; H, Nei′s genetic diversity; Na, observed number of alleles.

**Table 4:** Analysis of molecular variance (AMOVA) of five geographic regions.

Source of Variance	Degree of Freedom	Sum of Squares	Variance Component	Total Variation (%)
Among geographic regions	4	403.45	8.48	16.60
Within geographic regions	31	1320.13	42.58	83.40

**Table 5:** Nei’s original measures of genetic identity (above diagonal) and genetic distance (below diagonal) of five geographic regions *E. sibiricus*.

Population	SC	NM	XJ	GS	QH
SC		0.9460	0.9478	0.9192	0.6453
NM	0.0555		0.9552	0.8783	0.6170
XJ	0.0536	0.0458		0.9024	0.6514
GS	0.0843	0.1297	0.1027		0.7553
QH	0.4381	0.4829	0.4286	0.2807	

SC, Sichuan, China; NM, Inner Mongolia, China; XJ, Xinjiang, China; GS, Gansu, China; QH, Qinghai, China.

**Table 6:** Agronomic performance and the coefficients of variation (CV), mid-parent (MPH) and higher-parent heterosis (HPH) of hybrid population and parents.

Material Name	PH (cm)	LL (cm)	LW (cm)	FLL (cm)	FLW (cm)	CD (cm)	CN (No.)	TN (No.)	PL (cm)	AL (cm)	1000-SW (g)	SS (gf)	C.VV (%)
Y1005-1	56.3	12.6	0.9	9.1	0.8	0.2	3.1	183	20.0	1.1	4.4	41.9	18.37
ZhN06-1	98.0	16.2	0.8	9.1	0.6	0.4	3.4	152	19.2	1.1	2.4	97.2	17.09
F_1_-1	69.0	18.4	0.7	13.5	0.7	0.4	3.2	135	17.8	1.0	3.4	68.2	17.82
F_1_-2	79.0	20.2	1.0	15.7	0.9	0.4	3.3	111	20.0	1.1	3.5	93.0	16.03
F_1_-3	88.0	21.4	1.1	15.6	1.0	0.4	3.4	114	21.7	1.1	2.8	82.1	19.83
F_1_-4	82.0	22.6	1.1	17.5	1.1	0.4	3.1	95	23.3	1.3	5.1	114.9	14.46
F_1_-5	76.5	23.1	1.3	18.0	1.3	0.4	4.4	95	22.5	1.2	5.4	96.4	14.32
F_1_-6	81.7	22.8	0.9	18.8	1.1	0.3	3.7	132	19.4	1.1	1.8	143.4	16.82
F_1_-7	77.5	20.8	1.1	15.8	1.1	0.3	4.0	165	21.3	1.4	3.7	137.5	14.62
Max	98.0	23.1	1.3	18.8	1.3	0.4	4.4	183	23.3	1.4	5.4	143.4	19.83
Min	56.3	12.6	0.7	9.1	0.6	0.2	3.1	95	17.8	1.0	1.8	41.9	14.32
Mean	78.7	19.8	1.0	14.8	1.0	0.3	3.5	131.3	20.6	1.2	3.6	97.2	16.59
MPH (%)	2.5	48.4	21.8	80.9	44.0	13.3	10.3	-27.8	6.4	7.4	7.6	51.1	
HPH (%)	-19.3	32.0	10.0	80.4	23.5	-9.6	5.5	-33.9	4.2	6.9	-16.5	8.1	
SD	11.62	3.51	0.17	3.60	0.23	0.06	0.44	30.82	1.75	0.12	1.19	32.05	
CV (%)	14.78	17.74	17.28	24.32	23.55	17.47	12.61	23.47	8.50	10.28	33.11	32.98	

PH: Plant height; LL: Leaf length; LW: Leaf width; FLL: Flag leaf length; FLW: Flag leaf width; CD: Culm diameter; CN: Culm number; TN: Tiller number; PL: Panicle length; AL: Awn length; 1000-SW: 1000-seed weight; SS: Seed shattering degree; C.VV: C.V between varieties; MPH: mid-parent heterosis; HPH: higher-parent heterosis; SD: standard deviation.

**Table 7:** Results achieved by *Elymus* (Elw), *Pseudoroegneria* (Ps), *Leymus* (Lt) and *E. sibiricus* (ES) EST-SSR markers, all detected in two parents and seven F_1_s.

Primer ID	Bands Information					BSYF	BSZF	BEPP	BEPF
	T	M	TP	% P	PIC				
Elw0300s019	3	2	1	33.33	0.15	0	0	0	0
Elw0669s043	9	0	9	100.00	0.42	3	6	0	0
Elw1197s069	6	2	4	66.67	0.30	3	0	0	0
Elw1420s081	4	0	4	100.00	0.42	0	1	0	2
Elw1468s087	9	0	9	100.00	0.38	1	5	0	0
Elw1675s092	8	1	7	87.50	0.30	0	2	0	1
Elw2676s146	9	1	8	88.89	0.33	0	4	0	0
Elw2698s152	9	1	8	88.89	0.34	0	0	0	0
Elw2807s159	9	5	4	44.44	0.14	2	1	0	0
Elw2808s160	4	1	3	75.00	0.35	3	0	0	0
Elw3264s184	7	1	6	85.71	0.37	2	2	0	0
Elw3384s187	6	1	5	83.33	0.41	2	0	0	2
Elw3492s190	7	4	3	42.86	0.16	0	1	0	0
Elw3995s226	5	3	2	40.00	0.08	0	1	0	0
Elw4419s261	4	4	0	0.00	0.00	0	0	0	0
Elw5447s306	6	1	5	83.33	0.31	0	0	0	1
Elw5616s393	17	1	16	94.12	0.40	1	7	0	0
Elw5627s404	10	0	10	100.00	0.39	3	1	0	0
Ps2283	5	0	5	100.00	0.44	1	0	0	3
Ps261	9	3	6	66.67	0.25	4	0	1	1
Ps3447	5	4	1	20.00	0.04	1	0	0	0
Ps3577	4	2	2	50.00	0.10	0	1	1	0
Ps938	8	6	2	25.00	0.09	1	0	0	0
Ltc0209	8	1	7	87.50	0.31	2	2	0	0
Ltc0096	5	3	2	40.00	0.17	1	0	0	0
ES-7	3	3	0	0.00	0.00	0	0	0	0
ES-22	6	6	0	0.00	0.00	0	0	0	0
ES-23	4	3	1	25.00	0.05	0	0	1	0
ES-51	8	1	7	87.50	0.38	0	4	0	0
ES-97	4	4	0	0.00	0.00	0	0	0	0
ES-123	7	5	2	28.57	0.10	0	1	0	0
ES-125	5	5	0	0.00	0.00	0	0	0	0
ES-144	5	4	1	20.00	0.07	1	0	0	0
ES-231	4	4	0	0.00	0.00	0	0	0	0
ES-236	5	4	1	20.00	0.04	0	0	0	0
ES-253	11	6	5	45.45	0.13	1	0	0	1
ES-259	8	0	8	100.00	0.41	0	2	0	2
ES-310	7	7	0	0.00	0.00	0	0	0	0
ES-347	3	3	0	0.00	0.00	0	0	0	0
ES-405	1	1	0	0.00	0.00	0	0	0	0
Mean	6.4	2.6	3.9	59.92	0.20	0.8	1.0	0.1	0.3
Total	257	103	154			32	41	3	13

T, total number of amplified bands; M, number of monomorphic bands; TP, total number of polymorphic bands; % P, percentage of polymorphism; PIC, polymorphic information content; BSYF, bands shared by Y1005-1 and F_1_s; BSZF, bands shared by ZhN06-1 and F_1_s; BEPP, bands exclusively present in the parents; BEPF, bands exclusively present in F_1_s.

## References

[1] Dewey D.R. (1994). Cytogenetics of *Elymus sibiricus* and its hybrids with *Agropyron tauri*, *Elymus canadensis*, and Agropyron caninus. Bot. Gaz..

[2] Xie W.G., Zhao X.H., Zhang J.Q., Wang Y.R., Liu W.X. (2015). Assessment of genetic diversity of Siberian wild rye (*Elymus sibiricus* L.) germplasms with variation of seed shattering and implication for future genetic improvement. Biochem. Syst. Ecol..

[3] Ma X., Chen S.Y., Bai S.Q., Zhang X.Q., Li D.X., Zhang C.B., Yan J.J. (2012). RAPD analysis of genetic diversity and population structure of *Elymus sibiricus* (Poaceae) native to the southeastern Qinghai-Tibet Plateau, China. Genet. Mol. Res..

[4] You M.H., Liu J.P., Bai S.Q., Zhang X.Q., Yan J.J. (2011). Study on relationship of seed shattering, seed development and yield traits of *Elymus sibiricus* L.. Southwest China J. Agric. Sci..

[5] Wu J.F., Li F., Xu K., Gao G.Z., Chen B.Y., Yan G.X., Wang N., Qiao J.W., Li J., Li H. (2014). Assessing and broadening genetic diversity of a rapeseed germplasm collection. Breeding Sci..

[6] Jesske T., Olberg B., Schierholy A., Becker H.C. (2013). Resynthesized lines from domesticated and wild *Brassica*
*taxa* and their hybrids with *B. napus* L.: Genetic diversity and hybrid yield. Theor. Appl. Genet..

[7] Shen J.X., Fu T.D., Yang G.S. (2003). Relationship between hybrid performance and genetic diversity based on SSRs and ISSRs in *Brassica napus* L.. Agric. Sci. China.

[8] Zhao X.H., Jiang X., Zhao K., Zhao X.H., Yin J., Xie W.G. (2015). Screening of germplasm with low seed shattering rate and evaluation on agronomic traits in *Elymus sibiricus* L.. J. Plant Gen. Resour..

[9] Robins J.G., Bushman B.S., Jensen K.B. (2012). Dry matter yield combining ability among nine sources of orchardgrass germplasm. Euphytica.

[10] Jia X.P., Deng Y.M., Sun X.B., Liang L.J., Ye X.P. (2015). Characterization of the global transcriptome using Illumina sequencing and novel microsatellite marker information in seashore paspalum. Genes Genom..

[11] Ramu P., Billot C., Rami J.F., Senthilvel S., Upadhyaya H.D., Reddy L.A., Hash C.T. (2013). Assessment of genetic diversity in the sorghum reference set using EST-SSR markers. Theor. Appl. Genet..

[12] Xie W.G., Robins J.G., Bushman B.S. (2012). A genetic linkage map of tetraploid orchardgrass (*Dactylis glomerata* L.) and quantitative trait loci for heading date. Genome.

[13] Huang L.K., Huang X., Yan H.D., Yin G.H., Zhang X.Q., Tian Y., Zhang Y., Jiang X.M., Yan Y.H., Ma X. (2014). Constructing DNA fingerprinting of *Hemarthria* cultivars using EST-SSR and SCoT markers. Genet. Resour. Crop. Evol..

[14] Futuyma D.J. (1986). Evolutionary Biology.

[15] Yan J.J., Bai S.Q., Zhang C.B., You M.H. (2006). A primary investigation report for the wild germplasm of *Elymus sibiricus* in the Northwest Plateau of Sichuan province. Pruatacult Anim. Husb..

[16] Lei Y.T., Zhao Y.Y., Yu F., Li Y., Dou Q.W. (2014). Development and characterization of 53 polymorphic genomic-SSR markers in Siberian wildrye (*Elymus sibiricus* L.). Conserv. Genet Resour..

[17] Ma X., Zhang X.Q., Zhou Y.H., Bai S.Q., Liu W. (2008). Assessing genetic diversity of *Elymus sibiricus* (Poaceae: Triticeae) populations from Qinghai-Tibet Plateau by ISSR markers. Biochem. Syst. Ecol..

[18] Yan J.J., Bai S.Q., Zhang X.Q., You M.H., Zhang C.B., Li D.X., Zeng Y. (2010). Genetic diversity of wild *Elymus sibiricus* germplasm from the Qinghai-Tibetan Plateau in China detected by SRAP markers. Acta. Prataculturae Sin..

[19] Zhang J.C., Xie W.G., Wang Y.R., Zhao X.H. (2015). Potential of Start Codon Targeted (SCoT) Markers to Estimate Genetic Diversity and Relationships among Chinese *Elymus sibiricus* Accessions. Molecules.

[20] Schoen D.J., Brown A.H.D. (1991). Intraspecific variation in population gene diversity and effective population size correlates with the mating system in plants. Proc. Natl. Acad. Sci. USA.

[21] Stevens L., Salomon B., Sun G. (2007). Microsatellite variability and heterozygote excess in *Elymus trachycaulus* population from British Columbia in Canada. Biochem. Syst. Ecol..

[22] Slatkin M. (1987). Gene flow and the geographic structure of natural populations. Science.

[23] Schaal B.A., Hayworth D.A., Olsen K.M., Rauscher J.T., Smith W.A. (1998). Phylogeographic studies in plants: Problems and prospects. Mol. Ecol..

[24] Jin Y., Lu B.R. (2003). Sampling strategy for genetic diversity. Chin. Biodivers..

[25] Jensen K.B., Mott I.W., Robins J.G., Waldron B.L., Nelson M. (2012). Genetic improvement and diversity in Snake River wheatgrass (*Elymus wawawaiensis*) (Poaceae: Triticeae). Rangeland Ecol. Manag..

[26] Ozkan H., Levy A.A., Feldman M. (2001). Allopolyploidy-induced rapid genome evolution in the wheat (Aegilops-Triticum) group. Plant Cell.

[27] Levy A.A., Feldman M. (2002). The impact of polyploidy on grass genome evolution. Plant Physiol..

[28] Doyle J.J., Hewit G.M. (1991). DNA protocols for plants-CTAB total DNA isolation. Molecular Techniques in Taxonomy.

[29] Larson S.R., Kellogg E. (2009). Genetic dissection of seed production traits and identification of a major-effect seed retention QTL in hybrid *Leymus* (Triticeae) *wildrye*. Crop Sci..

[30] Mott I.W., Larson S.R., Jones T.A., Robins J.G., Jensen K.B., Peel M.D. (2011). A molecular genetic linkage map identifying the St and H subgenomes of *Elymus* (Poaceae: Triticeae) wheatgrass. Genome.

[31] Mott I.W., Larson S.R., Bushman B.S. (2011). Simple sequence repeat (SSR) markers for *Elymus*, *Pseudoroegneria* and *Pascopyrum* species (Triticeae: Gramineae). Plant Genet. Resour..

[32] Zhou Q., Luo D., Ma L.C., Xie W.G., Wang Y., Wang Y.R., Liu Z.P. (2016). Development and cross-species transferability of EST-SSR markers in Siberian wildrye (*Elymus sibiricus* L.) using Illumina sequencing. Sci. Rep..

[33] Zhao Y.F., Zhang X.Q., Ma X., Xie W.G., Huang L.K. (2014). Morphological and genetic characteristics of hybrid population of *Dactylis glomerata*. Genet. Mol. Res..

[34] Yeh F., Yang R., Boyle T. (1999). Quick User Guide. Popgene.

[35] Ghislain M., Zhang D.P., Fajardo D., Huamán Z., Hijmans R.J. (1999). Marker-assisted sampling of the cultivated Andean potato Solanum phureja collection using RAPD markers. Gen. Resour..

[36] Mengoni A., Bazzicalupo M. (2002). The statistical treatment of data and the analysis of molecular variance (AMOVA) in molecular microbial ecology. Ann. Microbiol..

[37] Zhang F.M., Ge S. (2002). Data analysis in population genetics. I. analysis of RAPD data with AMOVA. Biodivers. Sci..

[38] Pritchard J.K., Stephens M., Donnelly P. (2000). Inference of population structure from multilocus genotype data. Genetics.

[39] Evanno G., Regnaut S., Goudet J. (2005). Detecting the number of clusters of individuals using the software structure: A simulation study. Mol. Ecol..

[40] Rohlf F.J. (1992). NTSYS-pc: Numerical Taxonomy and Multivariate Analysis System.

